# Pneumonia and Hypokalemia Outcomes Investigated in a Rural, Midwestern Population

**DOI:** 10.7759/cureus.90247

**Published:** 2025-08-16

**Authors:** Mason P Penske, Claire E Reagen, Amanda M Sonnenburg, Megan A Unrath, Scott A Andelin, Nova D Beyersdorfer, Kerry D Johnson, John D Paulson

**Affiliations:** 1 College of Osteopathic Medicine, Kansas City University, Joplin, USA; 2 College of Osteopathic Medicine, Kansas City University, Kansas City, USA; 3 College of Osteopathic Medicine, Department of Primary Care, Kansas City University, Joplin, USA; 4 Family Medicine Residency, Freeman Health System, Joplin, USA; 5 Mathematics, Missouri Southern State University, Joplin, USA

**Keywords:** comorbidity, hospital mortality, hypokalemia, pneumonia, retrospective study, rural hospital

## Abstract

Background

Pneumonia is a broad term encompassing lung infections with varying causes, presentations, and prognoses. The objective of this study was to determine if the presence of hypokalemia in subjects admitted to the hospital for pneumonia was associated with an increased mortality rate compared to subjects admitted with pneumonia without hypokalemia. Finding comorbidities associated with worse outcomes in subjects with pneumonia could improve treatment and subsequently improve morbidity and mortality rates.

Methods

This retrospective study used data from the Freeman Health System (FHS) electronic medical record in Joplin and Neosho, MO, from January 1, 2019, to December 31, 2021. Hospital admissions of subjects ≥18 years old with pneumonia, hypokalemia, or both were identified through the International Classification of Diseases, Tenth Revision (ICD-10) codes related to pneumonia and hypokalemia. Of the 4,414 subjects with pneumonia, 1,045 concurrently had hypokalemia, and 3,369 did not. Additionally, 3,594 subjects had hypokalemia without pneumonia. Mortality rates of sample groups were assessed and compared.

Results

Of the sample groups identified, the mortality rate for subjects with pneumonia and hypokalemia was the highest at 218 (20.86%), followed by pneumonia without hypokalemia at 567 (16.83%), and hypokalemia but no pneumonia at 195 (5.43%). The two-sample comparison tests showed the differences in mortality rates in the three sample groups, which were all statistically significant.

Conclusions

The group with pneumonia and hypokalemia had a higher mortality rate than the group with pneumonia without hypokalemia or hypokalemia without pneumonia. This data suggests that hypokalemia was associated with increased mortality in subjects with pneumonia. This finding opens up discussion for identifying other comorbidities that may be present in subjects with pneumonia and could help decision-making in the care of subjects with pneumonia prior to hospitalization, upon admission, or during hospitalization.

## Introduction

Pneumonia is an umbrella term for infections affecting the parenchyma of the lungs. The etiology of the disease is variable, including bacterial, viral, and fungal pathogens. Pneumonia carries a heavy economic burden as one of the top 10 most expensive conditions in the inpatient setting [1]. One retrospective study from 2019 found initial admissions for community-acquired pneumonia (CAP) were associated with an average hospital stay of 5.7 days and a cost of $17,736 [2]. Hospitalization for pneumonia has been associated with an increased risk for developing cardiovascular disease in both short-term and long-term settings [3]. Subjects who do recover are at increased risk for long-term complications, including chronic respiratory conditions and neurocognitive impairment. Despite significant funding and research, efforts to improve mortality rates have been nominal [4].

There is wide variability in the severity of presentation and prognosis for pneumonia. This variability is related to multiple factors, including age, immunocompetency, vaccination status, and comorbid conditions [1,2]. Hypokalemia has been suspected to have some association with poorer outcomes in pneumonia for quite some time. In 1997, a retrospective analysis of subjects admitted to the hospital for bacterial pneumonia found hypokalemia might be a predictive factor for the severity of the disease course [5]. However, few studies have delved into the association of hypokalemia with morbidity and mortality in subjects with pneumonia. In 2020, a review found hypokalemia to be the second most common electrolyte abnormality present in CAP but did not find a predictive value for hospital readmission and pneumonia recurrence rates [6]. Another retrospective study in 2020 found an association between hypokalemia and increased severity of disease in subjects with COVID-19 pneumonia. Invasive mechanical ventilation was found to be needed significantly more frequently in subjects with COVID-19 who had hypokalemia, and hypokalemia was determined to be a highly sensitive predictor of worsening conditions in subjects with COVID-19 as well [7].

The objective of this retrospective study was to determine if the presence of hypokalemia in subjects admitted to the hospital for pneumonia was associated with increased mortality rates compared to subjects with pneumonia without hypokalemia.

The research data were previously presented by authors M. Penske, C. Reagen, and A. Sonnenburg in Joplin, MO, from April 4-7, 2024, at the Kansas City University Research Symposium in poster format.

## Materials and methods

This is a retrospective study with data collected from the Freeman Health System (FHS) electronic medical record (EMR) in Joplin and Neosho, MO, ranging from January 1, 2019, through December 31, 2021.

The sample included subjects ≥18 years of age (Table 1 for demographic information) who were identified by using the International Classification of Diseases, Tenth Revision (ICD-10) codes for pneumonia of various causes, hypokalemia, or both (Table 2), excluding those with prior relevant admissions. First, 5,128 subjects were identified as having ICD-10 codes for pneumonia. Of these, 714 subjects were excluded due to prior pneumonia admissions, resulting in 4,414 unique subjects with a pneumonia diagnosis (Table 2). Within this group, 1,045 subjects also had a hypokalemia diagnosis (P1), and 3,369 did not (P2). Additionally, 3,594 subjects were identified with hypokalemia without pneumonia (P3) as noted in Table 2.

**Table 1:** Demographic information for populations studied.

Population	Female	Male	18-64	≥65	African American	American Indian or Alaskan Native	Asian	Caucasian	Hispanic	Native Hawaiian or Other Pacific Islander	Other	Refused to Answer
P1	595	450	470	575	16	12	3	967	20	17	10	0
P2	1475	1894	1291	2078	38	30	14	3134	64	48	37	4
P3	2059	1535	1723	1871	55	34	16	3366	52	31	40	0

**Table 2:** Pneumonia and hypokalemia ICD-10 codes utilized in the study were obtained from the Freeman Health System electronic medical system (EMR) from Joplin and Neosho, MO, ranging from January 1, 2019, through December 31, 2021.

Pneumonia and Hypokalemia ICD-10 Codes	Diagnosis
J1000	Influenza due to other identified influenza virus with unspecified type of pneumonia
J1001	Influenza due to other identified influenza virus with the same other identified influenza virus pneumonia
J1008	Influenza due to an identified influenza virus with other specified pneumonia
J1100	Influenza due to unidentified influenza virus with unspecified type of pneumonia
J1108	Influenza due to unidentified influenza virus with specified pneumonia
J120	Adenoviral pneumonia
J121	Respiratory syncytial virus pneumonia
J122	Parainfluenza virus pneumonia
J123	Human metapneumovirus pneumonia
J1281	Pneumonia due to SARS-associated coronavirus
J1282	Pneumonia due to coronavirus disease 2019
J1289	Other viral pneumonia
J129	Viral pneumonia, unspecified
J13	Pneumonia due to *Streptococcus pneumoniae*
J14	Pneumonia due to *Hemophilus influenzae*
J150	Pneumonia due to *Klebsiella pneumoniae*
J151	Pneumonia due to *Pseudomonas*
J1520	Pneumonia due to *Staphylococcus*, unspecified
J15211	Pneumonia due to methicillin-susceptible *Staphylococcus aureus*
J15212	Pneumonia due to methicillin-resistant *Staphylococcus aureus*
J1529	Pneumonia due to another *Staphylococcus*
J153	Pneumonia due to *Streptococcus*, group B
J154	Pneumonia due to other streptococci
J155	Pneumonia due to *Escherichia coli*
J156	Pneumonia due to other Gram-negative bacteria
J157	Pneumonia due to *Mycoplasma pneumoniae*
J158	Pneumonia due to other specified bacteria
J159	Unspecified bacterial pneumonia
J168	Pneumonia due to other specified infectious organisms
J17	Pneumonia in diseases classified elsewhere
J180	Bronchopneumonia, unspecified organism
J181	Lobar pneumonia, unspecified organism
J188	Other pneumonia, unspecified organism
J189	Pneumonia, unspecified organism
J84116	Cryptogenic organizing pneumonia
J851	Abscess of the lung with pneumonia
J95851	Ventilator-associated pneumonia
E876	Hypokalemia

Next, 31,562 subjects were identified as having no pneumonia ICD-10 codes. Among these, 4,052 were excluded due to a history of prior pneumonia admission, resulting in 27,510 subjects without pneumonia (Table 2). From this group, a combined total of 23,916 admissions were excluded due to either a prior admission or absence of hypokalemia ICD-10 codes, leaving 3,594 subjects with hypokalemia but without pneumonia ICD-10 codes (P3) (Table 3).

**Table 3:** Sample group abbreviations used as a reference for the study. All sample groups were obtained through Freeman Health Systems EMR in Joplin and Neosho, MO, ranging from January 1, 2019, through December 31, 2021.

Sample Groups	
P1	Pneumonia with hypokalemia
P2	Pneumonia without hypokalemia
P3	Hypokalemia without pneumonia

The mortality rates of the groups were compared with sample proportions using Wald’s method. Subsequent two-sample proportion summary hypothesis testing with confidence intervals (CI) for the proportion difference was performed between each of the groups. All the tests were two-tailed, with a P <0.001 delineated as statistically significant. There were no post hoc tests employed. No power analysis was done as the data were collected directly from hospital records in a retrospective manner; thus, the sample size was fixed. The standard tests for comparing population proportions were used, and the raw formulas were programmed into Excel (Microsoft, Redmond, Washington).

## Results

**Figure 1:**
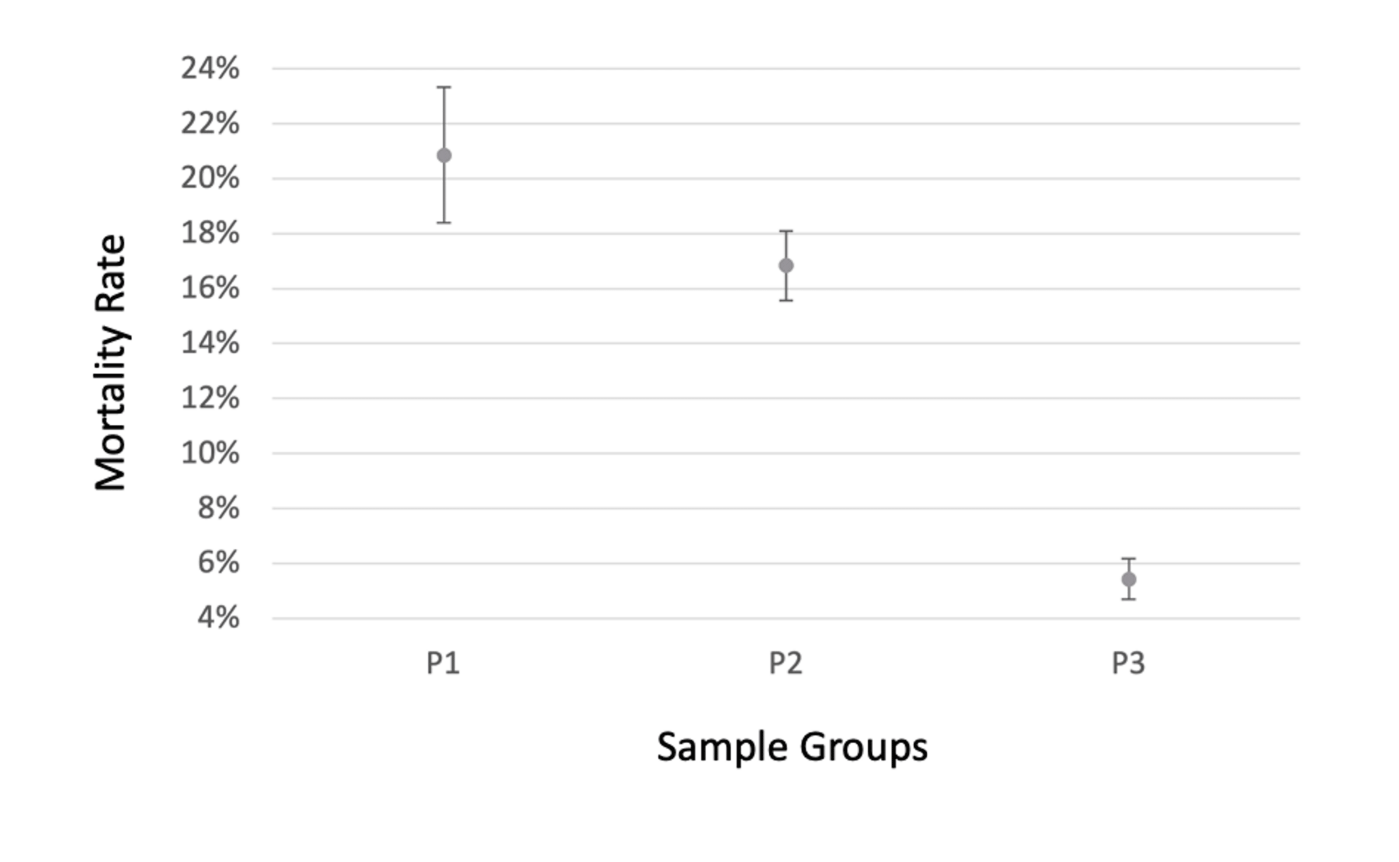
Mortality rate of the three sample groups. The Y-axis represents the mortality rate with the associated 95% confidence interval (CI). The X-axis represents the three sample groups, P1 (pneumonia with hypokalemia), P2 (pneumonia without hypokalemia), and P3 (hypokalemia without pneumonia) from Table 2.  A two-sample proportion test using Wald’s method was used to analyze the data.

In pneumonia with hypokalemia (P1), 218 (20.86%) subjects expired with a 95% CI between 18.40% and 23.32%. For pneumonia without hypokalemia (P2), 567 (16.83%) subjects expired with a 95% CI between 15.57% and 18.09%. Finally, for hypokalemia without pneumonia (P3), 195 (5.43%) subjects expired with 95% CI between 4.69% and 6.17% (Figure 1, Tables 4-5). The two-sample proportion test using Wald’s method was used to analyze the data between the three groups (P1, P2, P3)(Figure 1 and Table 4). The comparisons showed that the mortality rates between all three groups are significantly different (Table 5).

**Table 4:** Mortality and Confidence Intervals (CI) for individual sample proportions. The three sample groups are P1 (pneumonia with hypokalemia), P2 (pneumonia without hypokalemia), and P3 (hypokalemia without pneumonia) from Table 3.

Sample Groups	Mortality	Sample Proportion	Lower 95% CI	Upper 95% CI
P1	218 of 1045	20.86%	18.40%	23.32%
P2	567 of 3369	16.83%	15.57%	18.09%
P3	195 of 3594	5.43%	4.69%	6.17%

**Table 5:** Two-sample comparison tests with Confidence Interval (CI) results showing significant P-values. The three sample groups are P1 (pneumonia with hypokalemia), P2 (pneumonia without hypokalemia), and P3 (hypokalemia without pneumonia) from Table 2. A two-sample proportion test using Wald’s method was used to analyze the data.

Comparison	Mortality Sample 1	Mortality Sample 2	Sample 1 Vs Sample 2	Lower 95% CI for P1-P2	Upper 95% CI for P1-P2	P-value
P1 vs P2	218 of 1045	567 of 3369	4.03%	1.26%	6.80%	0.0029
	20.86%	16.83%				
P1 vs P3	218 of 1045	195 of 3594	15.44%	12.86%	18.01%	< 0.001
	20.86%	5.43%				
P2 vs P3	567 of 3369	195 of 3594	11.40%	9.94%	12.87%	< 0.001
	16.83%	5.43%				

Mortality in P1 was 4.03% higher than for P2, with a 95% CI between 1.26-6.80% (P=0.0029). Additionally, mortality in P1 was 15.44% higher than in P3, with a 95% CI between 12.86-18.01% (P<0.001). The mortality rate for P2 was 11.40% higher than that for P3, with a 95% CI between 9.94-12.87% (P<0.001) (Table 5). With all of the comparisons being statistically significant with P<0.05, this shows that P1 is associated with a higher mortality rate than P2 or P3. It also demonstrates that P2 is associated with a higher mortality rate than P3. Both of these findings indicate that pneumonia is the deadlier disease of the two, but hypokalemia as a risk factor in pneumonia is associated with an even greater increase in mortality.

## Discussion

This retrospective study analyzed subjects with pneumonia and/or hypokalemia in two hospitals in Joplin and Neosho, MO. When looking at the mortality rates of the three sample groups, subjects with pneumonia and hypokalemia (P1) had higher mortality rates than those with pneumonia but no hypokalemia (P2) and those with hypokalemia but no pneumonia (P3). Additionally, subjects with pneumonia without hypokalemia (P2) had a higher mortality rate than subjects with hypokalemia without pneumonia (P3). The sample comparisons showed that the mortality rates for all three samples were significantly different.

Overall, the data indicated that mortality for subjects with both pneumonia and hypokalemia (P1) was higher than for subjects who had one of the conditions without the other (P2 or P3), although the difference was less dramatic when compared to subjects with pneumonia without hypokalemia (P2). This suggests that the presence of hypokalemia is associated with increased mortality in subjects admitted to the hospital with pneumonia. In addition, it suggests that pneumonia is the deadlier disease of the two. There are likely other factors contributing to the mortality rates for subjects with pneumonia that were not accounted for in this study.

Pneumonia is a disease that can afflict anyone, but those with comorbidities are at serious risk. Apart from childbirth, pneumonia is the leading cause of hospital admissions for U.S. adults and the most common cause of death from infectious disease worldwide, surpassing both tuberculosis and HIV [1,8]. As of 2019, the American Thoracic Society estimates one million U.S. adults per year are treated for pneumonia in a hospital setting, with 5% of those subjects dying from the infection. Subjects who do survive the initial infection are still at high risk for morbidity and mortality, especially within the first year [4,9,10].

Pneumonia has been found to cause long-lasting cognitive deficits, initiating a tauopathy similar to Alzheimer’s and Pick's Disease. Cognitive impairment has been seen in a large minority of CAP survivors, regardless of age [4,11]. With the current understanding of the many comorbidities contributing to mortality rates in subjects with pneumonia, such as hypophosphatemia, hypocalcemia, hypokalemia, and hypoalbuminemia [10], this study can further open a discussion of early identification of subjects with pneumonia at risk for worse outcomes.

Limitations of this study include its retrospective nature; therefore, the sample was not chosen at random, and it cannot be determined if the sample is representative of the general population. This study examined hypokalemia as a potential risk factor. However, there may have been additional factors unaccounted for, which could have contributed to the significance of the mortality rate of the sample group with both pneumonia and hypokalemia. Other limitations include the subject population being limited to Southwest MO, indicating a specific geographic sample, and the possibility of human error in reporting in the EMR. Of note, within our study, the diagnoses of pneumonia and hypokalemia were not defined as present or not present on admission to the hospital.

## Conclusions

Our data demonstrated that subjects admitted to the hospital with both pneumonia and hypokalemia had higher mortality rates than those with pneumonia without hypokalemia and those with hypokalemia without pneumonia. This study confirms current literature about pneumonia and the importance of examining comorbidities, specifically with further insight into hypokalemia as a risk factor for increased mortality. Finding new and innovative ways to identify subjects at higher risk for mortality is imperative to improving healthcare and lowering pneumonia mortality rates. Going forward, further research could analyze how hypokalemia as a comorbidity interacts with other comorbidities and contributes to pneumonia mortality rates. This may help guide the management of subjects admitted to the hospital with pneumonia with specific comorbidities. Additionally, subjects at higher risk for worse outcomes with pneumonia can be identified sooner, possibly with the assistance of telehealth, by conducting preventative screenings of health and well-being. This may help providers counsel on better health decisions, such as smoking cessation and recommended vaccinations, as well as when it is appropriate to seek medical attention when there is suspicion of pneumonia.
